# Advancing Health Equity Efforts to Reduce Obesity: Changing the Course

**DOI:** 10.1146/annurev-nutr-092021-050805

**Published:** 2022-04-13

**Authors:** Shiriki K. Kumanyika

**Affiliations:** Dornsife School of Public Health, Drexel University, and Perelman School of Medicine, University of Pennsylvania, Philadelphia, Pennsylvania, USA

**Keywords:** obesity, health equity, public health, public policy, racism, social determinants of health

## Abstract

Population-based solutions are needed to stabilize and then reverse the continued upward trends in obesity prevalence in the US population and worldwide. This review focuses on the related, urgent issue of disparities in obesity prevalence affecting US racial/ethnic minority and other socially marginalized populations. The review provides background on these disparities from a health equity perspective and highlights evidence of progress in equity-focused obesity efforts. Five recommendations for advancing equity efforts are offered as potential approaches to build on progress to date: (*a*) give equity issues higher priority, (*b*) adopt a health equity lens, (*c*) strengthen approaches by using health equity frameworks, (*d*) broaden the types of policies considered, and (*e*) emphasize implementation science concepts and tools. Potential challenges and opportunities are identified, including the prospect of longer-term, transformative solutions that integrate global and national initiatives to address obesity, undernutrition, and climate change.

## INTRODUCTION

Obesity treatment is an ongoing clinical challenge, but the high prevalence and increasing severity in populations at large require public health solutions (85, 89, 148). This epidemic affects populations in the United States and globally with major economic, human, and societal costs (114). Many population-level drivers on obesity have been documented, and a substantial evidence base identifies many strategies to address them (50, 70, 148, 150). Available evidence supports policy, systems, and environmental (PSE) changes at macro and micro levels to be more supportive of healthy eating, physical activity, and weight control in information and marketing environments, schools, childcare, work, health care, and other public settings, and includes fiscal measures such as taxes on certain high-calorie foods and beverages. These approaches complement individually oriented education, counseling, and other informational approaches. However, the prevalence of obesity has continued to increase (118, 151) and reflects limitations in how to effectively implement and disseminate recommended approaches at a scope and scale large enough to have population-level impact, particularly on the drivers that are rooted in core societal processes (85).

**Figure 1:**
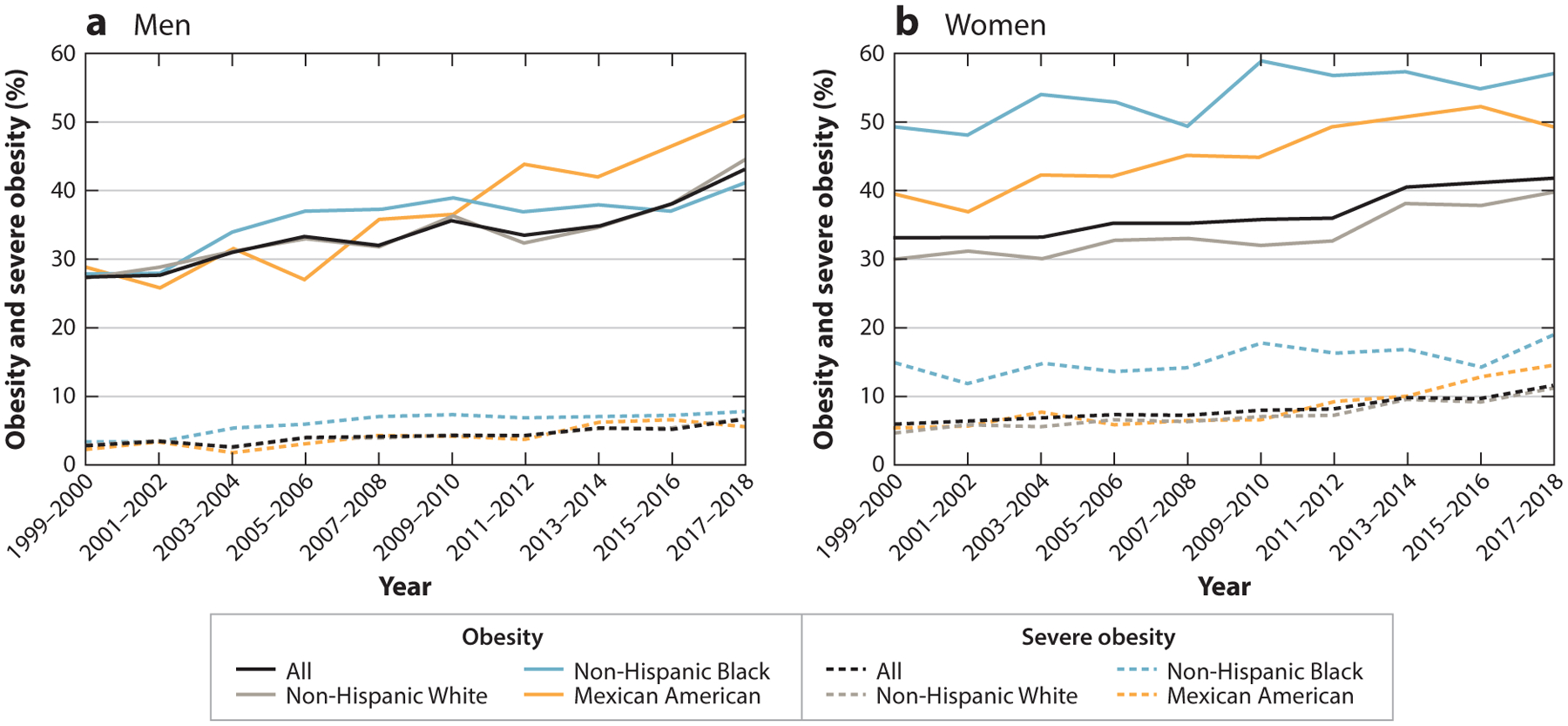
Age-adjusted prevalence of obesity and severe obesity in US adults between 1999–2000 and 2017–2018. Figure adapted with permission from Reference 118; copyright 2020 American Medical Association.

**Figure 2:**
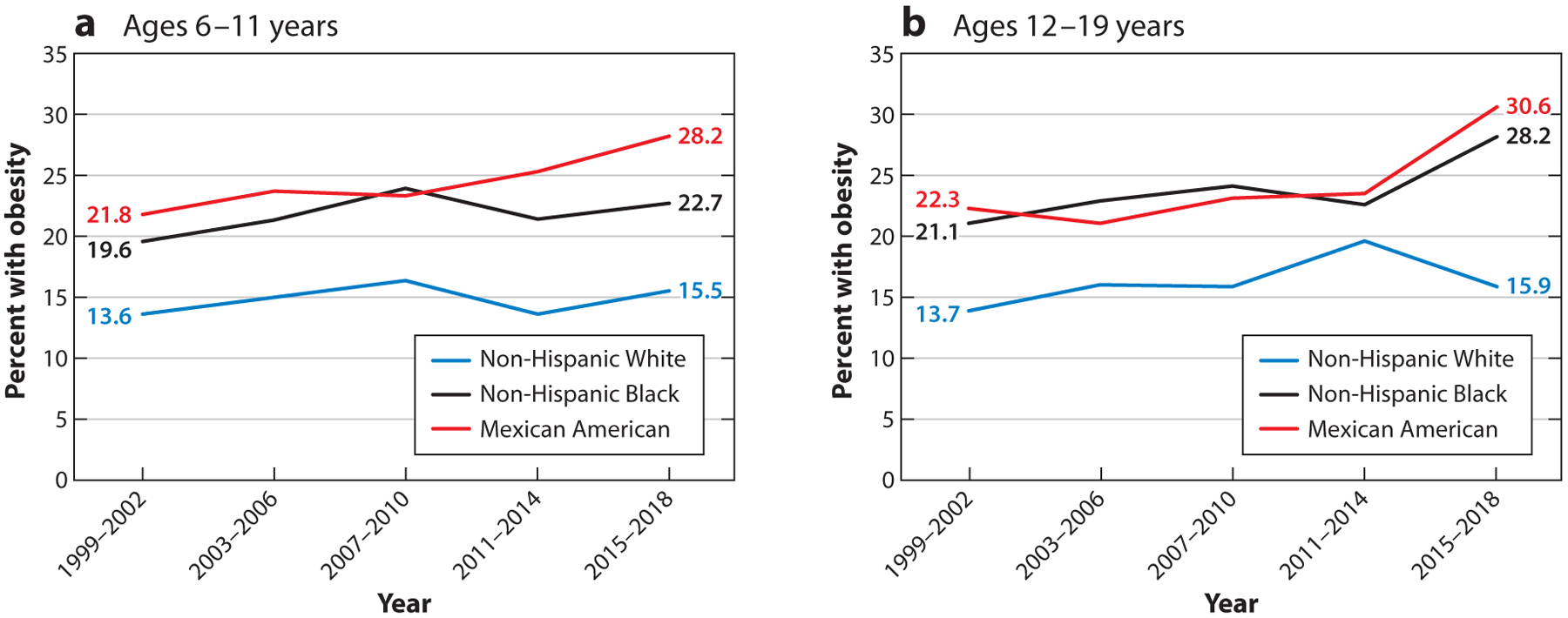
Prevalence of obesity in US children aged 6 to 11 years and adolescents aged 12–19 years, between 1999–2002 and 2015–2018. Figure adapted with data and permission from Reference 118.

US data show high and increasing obesity prevalence over the past two decades: from 30.5% to 42.4% in adults aged 20 years and older and from 13.9% to 19.3% in youth aged 2 to 19 years (29). In 16 states, 35% or more of adults have obesity, with the highest prevalence in the Midwest and South (29). Within this picture, disparities in obesity by racial/ethnic classifications are a prominent and persistent observation. Figures 1 and 2 demonstrate these disparities for Black and Mexican American men and women compared with White men and women and, with sexes combined, for children (118). Gender differences in obesity prevalence have been observed but may differ by developmental period and may also change over time as factors that contribute to obesity change (110, 127). Other data sources show that higher obesity prevalence is observed among Hispanic and Latino adults, other than Mexican Americans, and among American Indians/Alaska Natives, Native Hawaiians, and Pacific Islanders (108). With respect to Asian Americans, the standard definition of obesity [body mass index (BMI) ≥ 30] indicates significantly lower obesity prevalence compared with other populations (59). However, this definition of obesity is generally a less useful marker for obesity-related health risks in Asian Americans (107, 149). Patterning of equity issues varies among population groups and regions, although the types of issues are similar in concept.

This narrative review focuses on these racial/ethnic and related disparities, which must be addressed as a specific focus within the topic of population-wide obesity prevalence and trends. Treatment issues, although important, are not within the scope of this review. I primarily focus on data related to Black Americans, as there is more evidence about obesity and other health equity issues in Black people compared with others. I cite systematic and narrative reviews, original research, and commentaries selected to align with my sense of health equity issues in obesity solutions. Following an explanation of health equity concepts, I describe effects of racism as central to understanding the deep causes underlying inequities in populations of color as well as socioeconomic status (SES) effects. Highlighting evidence of progress in addressing equity issues in obesity, I recommend potential approaches to strengthen and build on these efforts and comment on challenges and opportunities.

## BACKGROUND

This section briefly reviews key concepts related to health equity reflected in US national health objectives and research guidance from the National Institutes of Health (NIH).

### Health Disparities and Health Equity

US public health authorities have recognized the need for addressing disparities, that is, disproportionately high rates of adverse health outcomes in racial/ethnic minority populations, in obesity, chronic diseases, maternal and child health, violence, and chemical dependence since at least the 1980s (112). The societal nature of the causes contributing to these disparities and the importance of addressing these causes were emphasized in the social determinants of health (SDOH) focus in the US Health Promotion and Disease Objectives for 2020 (80) and underscored in Healthy People 2030 (79). SDOH can be defined as broad social, economic, cultural, health, and environmental conditions and policies that affect living and working conditions; social, family, and community networks; and resources for managing health throughout the life course (22, 47). SDOH relate to inequities affecting a population group on average; that is, not all members of the group are similarly affected.

Health equity can be defined as the extent to which the right to health is achieved for and by people in groups that are discriminated against, marginalized, or excluded because of their race/ethnicity, social and economic circumstances, or other characteristics (22). Addressing inequities means correcting unfair circumstances—and this cannot be accomplished by treating people equally, as if everyone had the same starting points and opportunities; it invokes a moral argument. Also, the wealth of data indicating that health inequities are driven by social inequities directly refutes explanations of disparities in health as primarily of genetic origin (100) or as solely a matter of individuals’ behavioral choices (1). Duggan et al. (45) provide a thoughtful discussion of the problematic aspects of the use of race in (biological) scientific research—pointing out the numerous sources of variability that may contribute to ethnic variation in cardiometabolic risk. These authors endorse the concept that racism rather than race (see the sidebar titled Race versus Racism) should be viewed as the risk factor associated with people of color. Race as a biological construct has been discredited as a means of explaining health differences between populations of color (152)—groups commonly characterized as races, particularly in the US context—but the concept is still very much alive. Yet, while many scholars argue against the use of biological characterizations of race, some may argue that the concept is useful, and even essential, from a sociopolitical perspective to identify the problems caused by racism. The issue may be with how race is used. For example, Krieger (82) argues that measuring race without also measuring and analyzing the SDOH that race represents is problematic. In any case, the negative narrative about Black people persists through further elaboration associated with economic and political expediencies related to labor economics, housing and educational markets, and political power dynamics. The consequences, that is, social, economic, and health disadvantages affecting Black Americans, can be interpreted as evidence of inferiority.

Achieving health equity involves health in the absolute, that is, lowering disease or death rates, rather than only closing gaps in health outcomes between groups that are more or less advantaged. In the United States, White people are usually taken as the advantaged reference population representing the standard for good health. However, using the health of US White people as a standard is problematic. US health in general ranks at or near the bottom when compared with peer countries despite the highest spending on health care (128). The poor health of US White people reflects underlying problems with the US health-care system. Historically, and considering long-term global health trends, comparative World Health Organization and Organization for Economic Co-operation and Development statistics on mortality, morbidity, life expectancy, and risk factors for the highest ranked countries are understood to be acceptable indicators of health and health-care outcomes in best-case scenarios (115).

### Research Perspective: Framework of the National Institute of Minority Health and Health Disparities

**Figure 3:**
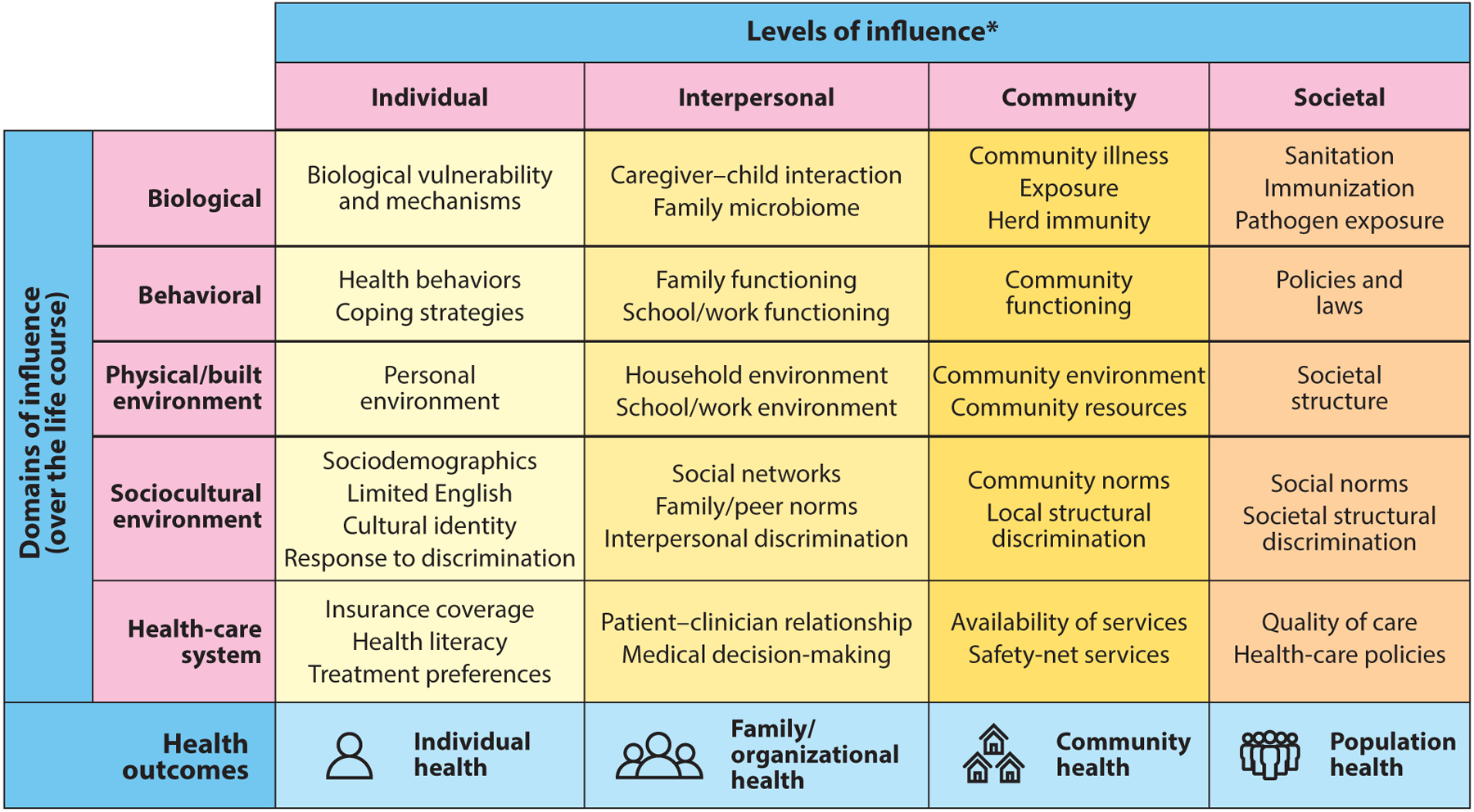
The NIMHD research framework that emerged to encourage research that addresses the complex and multifaceted nature of minority health and health disparities, including the examples of societal factors shown in the rightmost column. Abbreviations: NIMHD, National Institute on Minority Health and Health Disparities; SES, socioeconomic status. Figure adapted from NIMHD Research Framework (2018) (https://www.nimhd.nih.gov/docs/research_framework/research-framework-slide.pdf). National Institute on Minority Health and Health Disparities, 2018 *Health disparity populations: race/ethnicity, low SES, rural, sexual and gender minority Other fundamental characteristics: sex and gender, disability, geographic region

The NIH National Institute of Minority Health and Health Disparities (NIMHD) research framework is an important advance that establishes the legitimacy of NIH research on health equity issues (5) (Figure 3). It is annotated to identify priority populations for addressing disparities: race/ethnicity, low SES, rural residence, and sexual and gender minority status. The framework, which emerged from an NIMHD visioning initiative, exemplifies the recognition of adverse societal factors and associated SDOH as important contributors to disparities. It informs research funding at the NIMHD and the NIH overall. It emphasizes the value of including more than one domain and multiple levels in health disparities research (5), in contrast to the typically more narrow focus on individual level and biological and behavioral approaches.

Adaptations of the NIMHD framework for American Indian, Native Hawaiian, and Puerto Rican populations, posted on the NIMHD framework website (https://www.nimhd.nih.gov/about/overview/research-framework/), highlight the scope and nature of historical trauma and devastation from human rights abuses, colonization, cultural and family disruption, and other forms of legally authorized structural violence relevant to these populations. An adaptation for Black Americans would take note of transportation from the points of no return and enslavement of African people and their effects on culture and family life both during slavery and afterward in the Jim Crow era—the ongoing legacies of a system of legally authorized structural violence codified in laws related to civil rights, racial segregation, economic opportunity, and housing as well as criminal justice policies and practices (61, 93, 104).

## SOCIETAL AND SOCIAL DETERMINANTS OF HEALTH INEQUITIES

The term societal determinants refers to factors that are characteristic of and embedded in US society (e.g., racism, economic structures, governance systems, mainstream cultural values). They condition inequities that relate to the more specific social, economic, physical, and media contexts of day-to-day life.

### Race, Racism, and Socioeconomic Status

The premise here is that, with respect to people of color, race is not just one among many factors in a list of social determinants of health in the US context. Race is a fundamental marker of health disparities affecting Black and Brown people in the United States. Although Black and other populations of color are disproportionately affected by poverty and are less likely to have higher levels of education, racism effects lead to a much more complex picture of SES effects than might be recognized. It may be a common perception that low SES is the primary reason for racial/ethnic disparities and, when controlled in statistical analysis, will explain them. This may be the case in some instances but would be misleading or incomplete in others. Both racism-related and socioeconomic inequities matter to health (58), and the effects of these two types of influences are inextricably related for people of color.

Structural racism contributes to poor health through neighborhood or zip code effects, that is, limitations on access to high-quality schools or childcare opportunities and other services and opportunities, and lack of political voice associated with residential segregation (16, 17, 125, 145). Segregation is perpetuated by discrimination in housing, initially by legal means but now through covert lending practices that limit whether and where Black people can buy homes and the value of those homes. This, in turn, limits wealth building and perpetuates intergenerational poverty and financial instability. Residential segregation, together with income-related or transportation issues, may limit access to jobs. Policing and criminal justice policies and practices that criminalize Black men in particular remove potential wage earners from the community and limit the civil rights and employment potential of returning citizens. Segregation facilitates disinvestment in communities and schools in those communities and also limits political voice (16). Neighborhood racial composition also affects environmental risks such as exposure to pollutants, unsafe drinking water, and vulnerability to climate change (17, 102). Lack of quality early education can have negative effects on the entire educational trajectory and limit employment opportunities to lower-wage jobs without sick leave or health insurance benefits.

Thus, the marked Black–White disparities in selected socioeconomic indicators reported by Bailey et al. (16, 17) illustrate a combination of racism-related and socioeconomic inequities: a huge wealth gap (median assets of $110,500 versus $6,314 for White versus Black households); 10.1% versus 26.2% living below the poverty level; 12% versus 38% of children below age 18 living below the poverty level; 5.3% versus 11.3% unemployed; and 610 versus 3,611 per 100,000 males incarcerated (see table in 17). These socioeconomic disparities are correlated with health disparities related to infant mortality, years of potential life lost before age 75, and life expectancy and mortality related to heart disease and diabetes.

Evidence that a focus on socioeconomic variables alone can be misleading comes from observations that higher education and income do not necessarily result in equitable social opportunities for Black people compared with White people—a phenomenon that has been described as diminishing returns of high SES for Black Americans in association with several physical or mental health outcomes (11–13, 146). As an obesity-specific example, the Black–White gap in obesity prevalence among women is highest in the highest categories of income and education (117): The prevalence of obesity in White women decreases with increasing income and education from ~40% to ~27%, but this decline is not observed for Black women, among whom obesity prevalence is more than 50% in all income and education categories. The Black–White disparity widens from the low to high categories by ~15 to ~25 percentage points. Also, an analysis of data for people with very high incomes—defined as $175,000 or more annually—found that Black–White disparities were noteworthy, and greater than those of other minority populations, leading the authors to recommend against limiting studies of SES-related disparities to low-income populations. A qualitative study (67) of the diminishing-returns hypothesis suggests reasons why upward SES mobility might have smaller or even opposite health effects for Black and White people.

Although people of color are marginalized in other, predominantly White, high-income countries on the basis of indigeneity, regional origins (e.g., nationals of former colonies), or migrant status (88), what appears to be unique in the United States is the extent to which the society is race conscious and racialized in ways that are structural. Structural racism is embedded in national culture, economy, governance systems, and institutions that are interrelated in ways that are mutually reinforcing and adaptive and do not require actions by individuals with racist motives to maintain and perpetuate the racist character (145, p. 106). Racism is reflected in beliefs, policies, and actions that position White people as superior to Black people, where White refers to people of European descent. It is this concept of Black inferiority (and the attendant concept of White superiority) that makes one’s racial (ascribed) classification inescapable as an adverse influence on one’s social, economic, and political condition.

### Pathways Between Social Determinants and Obesity

The foregoing discussion of SDOH has clear relevance to obesity but is not obesity specific. Table 1 provides an overview of how SDOH influence obesity development and interventions. It highlights SDOH and related factors for which there is largely consistent evidence linking inequities in physical, economic, policy or political, and sociocultural environments to inequities in obesity affecting US Black people and other populations of color (15, 39, 55, 56, 70, 74, 84, 91, 98, 123, 135). Specific pathways may be altered by changes in these environments. The format follows Swinburn et al.’s (135) ANGELO model for analyzing environments that contribute to obesity, co-occurring in place and time; this model has been applied to the US obesity epidemic as a whole to identify action priorities (68). As shown, SDOH have direct influences on obesity inequities through several types of food-related and physical activity factors and, from a policy perspective, also through policies that directly or indirectly affect these factors.

**Figure 4:**
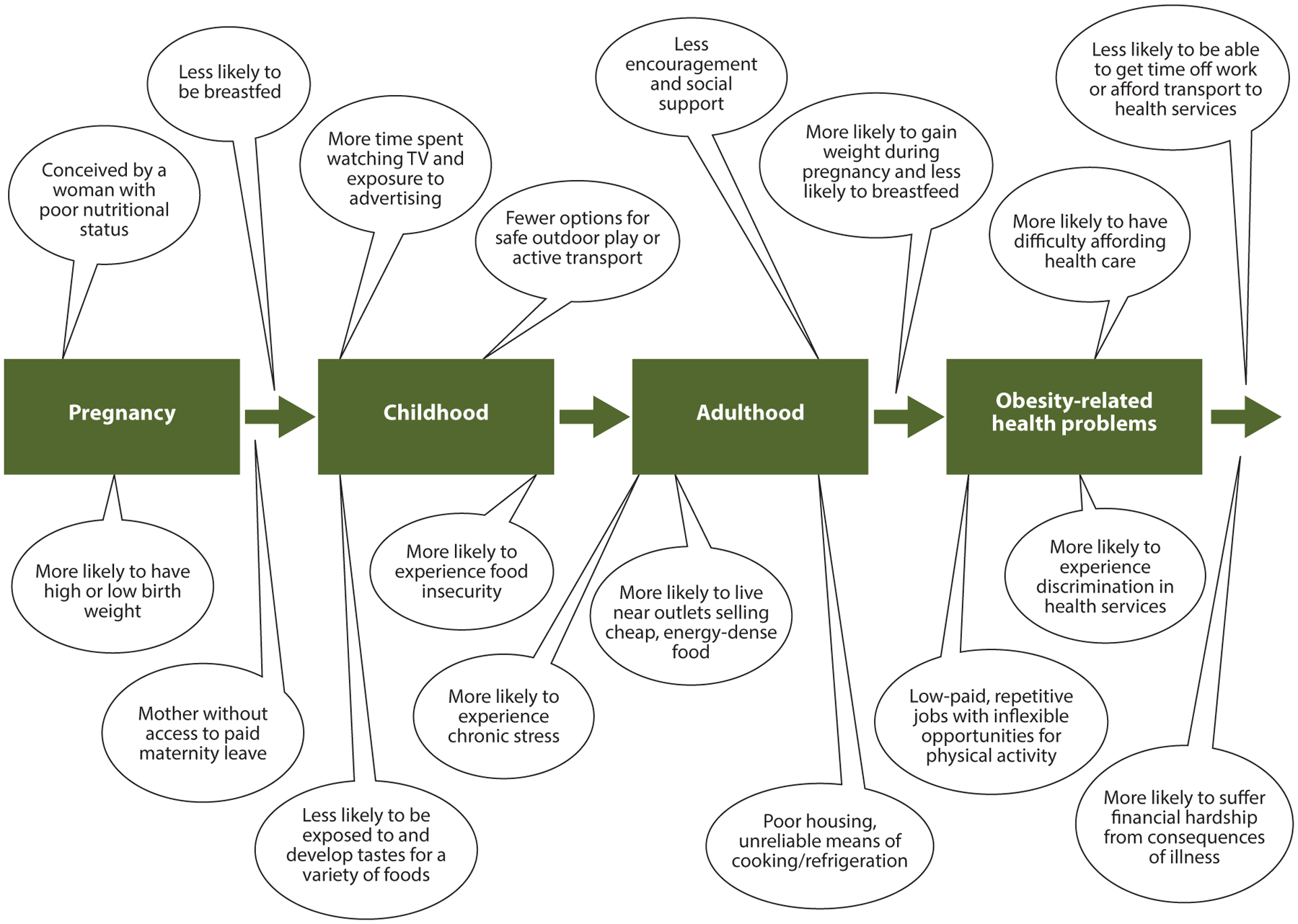
How inequities in obesity compound over the life course. Adverse social and economic conditions that begin before or during pregnancy or arise over time can have cumulative effects on risks of obesity, and related health problems are potential targets for policy solutions. Figure adapted with permission from Reference 97.

Figure 4 depicts ways that social inequities linked to nutrition and obesity-specific exposures compound over the life course (97). The inclusion of exposure to chronic stress reflects its role as an important potential mediator of the adverse environmental influences illustrated in Table 1 (71, 83). Chronic exposure to stress and trauma and poor sleep or short sleep duration, which are interrelated, are also relevant pathways whereby SDOH affect obesity (7). However, as a caveat, a concern is that representations of adverse exposures may ignore the personal or community assets that can be brought to bear in coping with various stressors. For example, sociocultural factors may include survival skills honed across generations of exposure to adversity (resilience) as well as positive health behaviors retained from cultures of origin. Recognizing such assets is important for countering the excessively negative, deficit-oriented perceptions of populations of color. Purely negative representations are too often promoted by the prevailing narrative and are also inadvertently perpetuated by repeated, decontextualized descriptions of disparities and their causes.

## EVIDENCE OF PROGRESS

In the interim before prevalence stabilizes and then declines with some consistency, discussions of progress must consider where current approaches appear to be on the right track. Applying this observation to equity-focused efforts, we would consider approaches of interest to include interventions that reduce prevalence in a racial/ethnic minority or low-income population in the absolute and relative to the reference population. As reviewed below, there is some promising evidence related to policy-based changes associated with the Healthy Hunger Free Kids Act (HHFKA) and from evaluations of community-wide approaches.

### Supplemental Nutrition Program for Women, Infants, and Children

Women and children in low-income households are eligible for the Supplemental Nutrition Program for Women, Infants, and Children (WIC) (141). This program reaches millions of eligible people annually, although only about half of those eligible, i.e., ~6.7 of ~12 million, participated per month in 2018 (140). The WIC benefit levels, foods, support for breastfeeding, and implementation guidelines were revised beginning in 2009 along the lines of guidance from an Institute of Medicine report (69). Data from more than 12 million two-to-four-year-old children who received WIC benefits between 2010 and 2016 showed a consistent pattern of small declines by age, gender, and race/ethnicity; declines were significantly greater in almost all racial groups relative to White people (121). During the same period, National Health and Nutrition Examination Survey (NHANES) data for two-to-five-year-olds indicated that previously declining trends in children were increasing by 2015–2016 in the general US population (60). The 2017–2018 NHANES data show no significant increase in obesity prevalence in any racial/ethnic group sampled in children aged birth to five years, in contrast to increased prevalence in school-aged children and adolescents (118). Thus, WIC—with its wide reach—may have contributed to stabilization of obesity prevalence trends in the youngest children (40). A reported decrease in obesity prevalence among children in Los Angeles County (primarily Hispanic) after implementation of the new food packages (33) also supported a positive WIC impact. The Los Angeles finding was not modified by income or poverty level (34) but differed among girls (less risk reduction than for boys) living in neighborhoods with a high density of unhealthy food outlets (8).

### School Meals

Positive effects of changes in school meal dietary quality on obesity have been reported as well. The National School Lunch Program reaches approximately 30 million children from low-income families on an average school day (142). Kinderknecht et al. (78) estimated improvements in dietary quality from a series of cross-sectional analyses of NHANES dietary recall data for 15-to-18-year-old participants and nonparticipants in the school lunch program before (2007–2010) and after (2013–2016) HHFKA changes in meal guidelines. Dietary quality, assessed with the 2010 Healthy Eating Index, for lunch and over the entire day increased among program participants by several percentage points compared with nonparticipants over the same period. The School Nutrition and Meal Cost Study (SNMCS), which provides complementary data based on menu analyses of breakfasts and lunches served in a representative sample of US schools, also indicated a substantial improvement in meal nutritional quality after implementation of the new guidelines (53). Other SNMCS analyses explored but did not find income or racial/ethnic disparities in the nutritional quality of meals (18).

Kenney et al. (76) estimated effects of school meal changes on obesity trajectories of 173,013 10-to-17-year-old children represented in the National Survey of Children’s Health, with 2003–2012 as the reference period and 2016–2018 as the postimplementation period. The noteworthy finding was a 47% lower obesity prevalence below the projected rate by 2018 among children from families below poverty (an estimated 500,000 fewer cases of obesity among children living in poverty), in contrast to no effect in children living above poverty. Whether these promising results will continue and prove to be sustainable may depend, among other things, on retention of the strong guidelines initially established (38).

### Retail Food Access and the Supplemental Nutrition Assistance Program

Food retail environment interventions address certain private sector issues related to food stores, food businesses, and food marketing but do not necessarily affect retailing practices or dietary quality of consumer food purchases. Retail environments are critical for influencing household food purchases of participants in the Supplemental Nutrition Assistance Program (SNAP), formerly known as the Food Stamp Program, which is the largest US federal nutrition assistance program (143). Most benefits are used in conventional supermarkets or large grocery stores. SNAP benefits are equity focused because they address the income gap in the ability to purchase food. However, they are not nutrition targeted. For reasons that date to the program origins, the income assistance objectives are prioritized and there are relatively few exceptions to types or amounts of foods that can be purchased.

A comprehensive review by Moran and colleagues (105) identified evaluative data from simulations, experimental studies, or natural experiments for seven policy actions related to food retail, grouped into three categories: (*a*) standards related to nutrition labeling and health claims (calorie labeling of prepared foods in supermarkets); (*b*) economic approaches (SNAP benefit increases or SNAP financial incentives for healthy food purchases, taxation of sweetened beverages, and WIC food package revisions); and (*c*) policies to improve retail food environments (financial support for supermarkets to locate in underserved areas and rules allowing SNAP purchases online). Some of these policies have major equity implications in that they relate to income-targeted programs such as WIC and SNAP and to disincentives to consume sweetened beverages, for which Black people have higher marketing exposure and consumption (63, 92). The clearest findings favorable to a policy impact on obesity prevention were those associated with the WIC food package changes, sweetened beverage taxes as a deterrent to their purchase, and the incentive-based increase in fruit and vegetable purchases. However, Moran et al. note that policy effects on racial/ethnic or income disparities were not addressed in the studies reviewed.

Funding for new supermarkets or grocery stores, a highly visible federal as well as state and local policy approach, has been linked to improvements in food security but not dietary quality—despite its designation as a Healthy Food Financing Initiative (99). The same was true for assessments of an increase in SNAP benefits. This may reflect the fact that most food purchases, including those purchased with SNAP benefits, are used in supermarkets or grocery stores for which the in-store retailing format is not conducive to healthy food purchases (99). Mah and colleagues (99) point out that in-store retailing interventions have generally emphasized information and education, ignoring the larger retail policy contexts related to retailer or food company behavior.

### Community-Wide Efforts

Liao et al. (95) reported statistically significant reductions in obesity prevalence in Black adults in communities engaged in Centers for Disease Control and Prevention (CDC)-funded programs that focused on cardiovascular disease or diabetes. These programs were funded in 2010 through the CDC’s program on Racial and Ethnic Approaches to Community Health (REACH). REACH grantees work directly with and in communities to identify and implement strategies that are best fits for community assets, resources, and needs (30). Impact on obesity was assessed by comparing annual survey data from these communities with propensity-score matched Behavioral Risk Factor Surveillance System data for White and Black adults in other communities in the same states. Obesity prevalence declined by about 1 percentage point per year in the REACH communities, for an overall decrease of 2.1 percentage points compared with an increase of 1.0 percentage points in other communities (relative decline of 5.3 and 2.4 in the comparative data), with a similar pattern for severe obesity—both effects were statistically significant (95).

Case studies of four cities where declines in obesity had been observed also support the effectiveness of comprehensive community-wide initiatives (44, 72, 120). In Philadelphia, Pennsylvania, for example, greater declines in obesity were observed in Black and Latino children compared with White children. A wide array of policies and programs were implemented in the period during which obesity declined (42). These programs were funded by various federal grants and local initiatives, and many were longstanding efforts. The Philadelphia efforts were implicitly and explicitly targeted to high-risk communities: Nearly 60% of the Philadelphia population is Black or Latino (139), and Philadelphia has the second-highest percentage of people living in poverty of the 10 largest US cities, at 23.3% (133).

The Healthy Communities Study, conducted between 2010 and 2016, observed mixed effects of obesity efforts in 130 US communities (86). The objective of this observational study was to obtain a real-world picture of how US communities had responded to the childhood obesity epidemic. Current and prior community policies and programs (CPPs) were assessed through document reviews and key informant interviews. BMI data were collected directly with a longitudinal perspective obtained from height and weight data in medical records. An explicit equity focus was also built into the sampling and measurement design, to permit subgroup or effect modification analyses by race/ethnicity, income, and region. The main analysis results indicated that implementation of CPPs consistent with recommended strategies had a cumulative, positive effect on children’s BMI trajectories. However, the positive effects were limited to White children or communities in families with above-median incomes in the US Northeast—implying a widening of inequities (134). The lack of positive effects among Black and Latino children and communities or among families with lower incomes indicated a widening of gaps.

Relevant to community-wide approaches, the gradual adoption of systems thinking and applications in the obesity field, including in relation to health equity (10, 52, 74, 129), has been promising. Toward this end, the multisector National Academy of Medicine Roundtable on Obesity Solutions has been engaging its members in internal and public discourse that considers ways that systems science can be used in obesity prevention and control efforts. This initiative includes a pivotal focus on equity issues such as structural racism (109). Greater use of systems science will be critical in the long term.

### Additional Findings

Several systematic reviews address the effectiveness of various approaches on socioeconomic disparities in healthy eating, physical activity, or obesity in the United States and elsewhere. A beneficial effect on disparities was typically defined as selectively improving outcomes for people with low SES or having no difference in effects by SES, versus a deleterious, gap-widening effect of approaches with benefit only among those with higher SES (21, 25, 103, 119). McGill et al. (103) reported findings supporting positive effects on equity for upstream or structural approaches versus a gap-widening effect associated with education and counseling approaches. Boelsen-Robinson et al. (21) reported a consistent, positive finding (9 of 10 studies) that whole-of-community studies resulted in better or similar effects on body weight for subgroups with low versus high socioeconomic position. Interventions with positive findings typically included at least two of the following: changes to environments, addressing more than three settings, or community engagement, as well as evidence of considering equity in the study design. Olstad et al. (119) reported a consistent finding that comprehensive school-based policy approaches, and fruit and vegetable subsidies, were effective for children, but they did not find effects of government policies in adults.

With respect to racial/ethnic disparities, Flórez et al. (49) reported that faith-based interventions may be the most well studied and promising for multilevel, community-partnered approaches with the potential to be scalable and sustainable. Based on their review, the authors found that US studies are primarily with Black communities, along with some in Latino communities, and provide sufficient evidence on which to build future initiatives. Faith organizations have intrinsic characteristics that align with and may define cultural and other aspects of community contexts, and some provide services that reach beyond their congregants. Faith-based approaches have the potential to address multiple socioecologic levels, although those evaluated to date have not necessarily taken full advantage of the opportunities to do so. The sustainability of faith-based approaches has not been well studied.

Other reviews of interventions with Black or Latino populations are more critical of the evidence base than informative about potentially effective approaches. A set of 10 systematic reviews of obesity or related interventions in Black adults and children published up to about 2012 concluded that the small number of studies identified was very heterogeneous and of low quality in many respects (91). A subsequent review of obesity disparities research by Pratt et al. (124) that focused on studies published between 2011 and 2016 also found the evidence base to be of limited size and quality. Limitations of the evidence base have also been noted by the US Preventive Services Task Force. The task force guideline authors note that it was “not possible to make conclusions about whether the health effects of weight loss interventions varied according to baseline BMI category, age, and race/ethnicity” (94, p. 1185) and recommend further research in diverse populations.

Haughton et al.’s (64) review of inclusion of racial/ethnic groups in behavioral weight loss interventions from 2009 to 2015 confirmed the need for more studies inclusive of people from diverse racial/ethnic groups and explicit attention to the potential for differential outcomes by race or ethnicity. Alcántara et al. (3) examined both representation and attention to context in data from a comprehensive review of studies using self-regulation approaches to address a variety of behaviors. These authors also reported limited representation of racial/ethnic minority populations in behavioral intervention studies and found that relatively few analyzed effect modification by race/ethnicity or social context variables. Dietz (43) emphasized the importance of addressing community contexts in a commentary on the null results of five major NIH-funded obesity prevention trials that targeted Black or Hispanic children or children from underserved communities—trials that were otherwise well designed and implemented but did not give sufficient consideration to addressing adverse exposures in the larger community contexts in which they were conducted.

Overall, what seems to selectively address disparities are policy approaches such as WIC and school meals, which provide food to low-income households, and well-designed and implemented whole-community or community-engaged interventions with predominately low-income or minority communities or in faith settings. What does not seem to work at a level that would result in substantial equity impact is translating lifestyle behavior change interventions in community settings without a systems approach that also addresses multiple contexts and socioecologic layers.

## ACCELERATING PROGRESS

**Figure 5:**
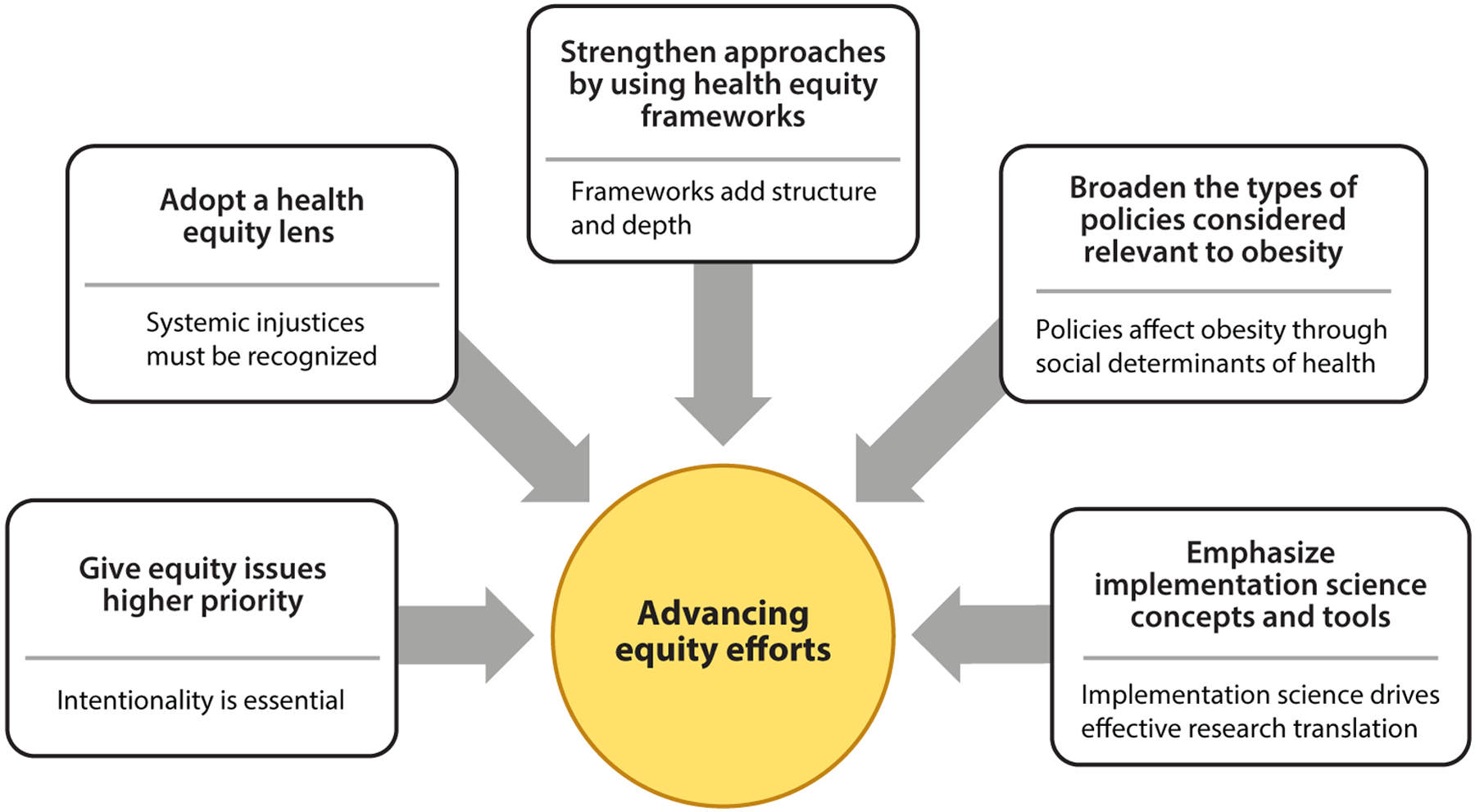
Five recommendations for advancing equity in obesity efforts. These actions emphasize the need for greater priority and understanding of systemic health equity issues and the varied policies that influence obesity risk and management. Implementation science offers approaches for identifying effective and sustainable solutions.

We know what is recommended on the basis of evidence-informed policies and programs (70, 77, 148, 150). Yet we are uncertain about how to implement these policies and programs to ensure their effectiveness and sustainability, and we are particularly uncertain about how they fit diverse communities. We know that approaches that do work will have to work better in high-risk populations to close gaps. We are concerned that disparities may be widening because of inabilities to translate evidence-based interventions to communities experiencing social and health disadvantages. This section presents five recommendations, highlighted in Figure 5, with the potential to accelerate progress. The recommendations are numbered below only for ease of reference—all are of high priority.

### Recommendation 1: Assign Higher Priority to Addressing Health Equity Issues in Research, Policy, and Practice

Accelerating progress in equity-focused obesity efforts will only benefit from any progress in society at large if more equity-relevant interventions are undertaken and appropriately targeted and tailored (see the sidebar titled Targeting and Tailoring). It is difficult to overstate the importance of an intentional focus on equity. Insufficient priority for equity issues may fail to close gaps and may even widen them. Intentionality sets the tone for the entire process of policy or program analysis, design, or redesign, as well as implementation and assessment of outcomes. It challenges the notion that any well-designed policy or program would, or should, work in the same way for everyone. Attention to equity issues requires routinely examining general guidance for how contextual issues of different populations have been considered and, where necessary, using approaches that target and tailor accordingly.

Recommendations to increase use of targeted and tailored policies may meet with resistance on the basis of misperceptions that one size does or should fit all, perceived lack of efficiency, biases about the deservedness of certain population groups, disbelief that targeted approaches can be fair or effective, or perceptions that singling out a particular ethnic group is inherently stigmatizing (57). Therefore, giving priority to health equity requires an explicit statement of the relevant principles and measures, with strong justification, to ensure that policy implementation is not subject to discretionary introduction of inequities on the basis of the perceptions and biases of policy actors downstream (57, 153). In the behavior change field, resistance to targeting and tailoring of intervention approaches may be influenced by issues of fidelity versus fit that raise concern about altering approaches found to be efficacious under controlled conditions (27). Or, contextual issues that would trigger targeting or tailoring may be recognized but viewed as not in scope for relatively short-term behavioral approaches that focus on self-regulation (132, p. 535).

### Recommendation 2: Adopt a Health Equity Lens

Everyone views the world through various lenses on the basis of, for example, professional training, social position, and personal experiences and values. Adding a health equity lens to obesity efforts focuses on the ability to recognize where and how contemporary injustices or legacies and lack of resources or opportunity affect health-related behaviors and outcomes. An equity lens brings to the foreground contextual factors that may be taken for granted as status quo or overlooked as influences on obesity-related behaviors. It calls attention to societal forces and SDOH, including power dynamics, community voice, and access to decision makers who can effect change.

Applying critical race theory (CRT) underlies the concept of an equity lens (51, 65). As articulated by Ford & Airhihenbuwa (51), core concepts include the explicit acknowledgment of the role of racism as a dominant force in US society, the role of critical consciousness to develop an in-depth understanding of the manifestations of racism and its historical roots, and the structural forces that perpetuate racial bias in research and among researchers. Intersectionality is also core to the CRT vocabulary, referring to the intersections of race, social class, and gender, for example, such that these identities, in combination, determine the way a person is viewed and treated in society. Dimensionality, as articulated by Hogan et al. (66), emphasizes the importance of grounding considerations of race within historical and life-course perspectives—recognizing that adverse exposures and outcomes start in utero and that the ability to benefit from an intervention will be constrained by multiple adverse experiences along the life course: hardships and various crises that may sabotage efforts to cope on a day-to-day basis and have cumulative adverse health effects (see Figure 4).

Community engagement is a critical aspect of an equity lens. Effective PSE strategies work with community stakeholders and within contexts to facilitate relevance, fit, receptivity, and uptake of policies and programs. The Committee on Accelerating Progress in Obesity Prevention emphasized that a high level of engagement with community leaders, residents, and other stakeholders is required to plan and implement their recommended PSE strategies in ways that address equity issues with carefully targeted approaches (70). Levels of community engagement can be described along a spectrum from limited outreach, to consultation, involvement, and collaboration, to—maximally—shared leadership characterized by strong, bidirectional relationships (37). Not all community engagement processes will attempt the level of community engagement that maximally adheres to all underlying principles. However, reference to the continuum is useful for thinking through what level is appropriate and what process will be used (113).

### Recommendation 3: Employ Health Equity Frameworks and Tools

Advancing health equity efforts requires equity frameworks that facilitate identification of intervention elements and pathways to guide hypotheses about what might work or to use retrospectively to understand why a given intervention or set of interventions did or did not have the intended effect on equity. Useful frameworks may relate to research, policy or program design, adaptation, implementation, delivery setting, population of interest, and relevant environmental contexts.

Health equity frameworks can be applied to eating, physical activity, and obesity research and practice. As such, they shift or expand the perspective from obesity or energy balance as the entry point to health equity, while considering both perspectives. Health equity frameworks amplify structural issues and SDOH, while the obesity perspective provides important content. This also applies to other diet- and physical activity–related chronic disease–related outcomes such as diabetes or cardiovascular diseases. Health equity efforts intrinsically leverage concepts such as fairness and social justice to argue for and motivate public and political will for structural changes. Moreover, pursuing equity in obesity from a broader health equity perspective fosters synergy; it may lead to collaborations combining SDOH-related initiatives that focus on obesity with those related to other health outcomes. Besides being predicated on the previously described health equity concepts and principles, health equity frameworks provide tools for assessing policies and programs by prompting for deconstruction and reconstruction of intervention and contextual elements on the basis of a series of questions or list of factors to consider.

Health equity frameworks are designed to operationalize health equity concepts for various purposes, as follows: integrating behavioral interventions and health equity approaches or principles (3, 4); disparities reduction in health care and health equity research and practice (35, 96, 131), including e-health (9); frameworks focused on study design, analysis, and reporting, including for systematic reviews (75, 122, 144); design of multilevel interventions based on the NIMHD framework (2); guidance for policy analysis (14, 19, 26, 74, 153); and frameworks to guide implementation science approaches (6, 32, 41, 46, 106, 116, 138, 147). Selected frameworks and tools of particular relevance to obesity are highlighted in Table 2 (28, 31, 32, 36, 41, 56, 87, 90).

### Recommendation 4: Broaden the Range of Policies Considered as Relevant to Obesity

An NIMHD working group paper points to the need for more evidence on the effectiveness of structural interventions for eliminating disparities (23). They characterize structural interventions as disease agnostic in their focus on altering SDOH-related policy or other contexts and cite examples showing how structural interventions that focus on housing, early childcare, and income supports are relevant to obesity. Joshi et al. (74) offer a policy equity assessment methodology for analyzing the logic, capacity, and evidence for outcomes of policies with respect to health equity issues—some may close gaps, have neutral effects on equity, or widen gaps. Using Section 8 housing policies, Head Start, and the Family Medical Leave Act, they point out how policies affecting the same population group may have very different designs and provisions that affect access and coverage, reporting requirements, and accountability in terms of monitoring. Thus, there is utility in joint consideration of policies with complementary, overlapping, or conflicting effects on obesity through effects on SDOH.

### Recommendation 5: Emphasize Equity-Focused Implementation Research

This recommendation offers a path forward for utilizing the perspectives and approaches described in the prior recommendations. Advances in the visibility of implementation science and methods offer the permission and potential to move beyond approaches in which evidence-based interventions (EBIs) based on tightly controlled randomized controlled trials are taken as the gold standard for practice in the real world (24, 101). This area of science responds to the need for better and more timely translation of research evidence to population-level applications and is founded on principles such as adaptation to settings and contexts, external validity, multidisciplinary approaches, stakeholder engagement, and measurement of variables indicative of both process and outcomes at multiple levels. Dissemination and sustainability are also core to knowledge translation. The above-cited frameworks provide guidance for systematic application. Moreover, implementation science is also highly compatible with systems thinking and systems-oriented approaches.

The applicability of implementation science to obesity research relates to the fact that eating and physical activity behaviors are embedded in everyday life settings that affect intervention effectiveness. Applicability to health equity research relates to the fact that adversities of day-to-day living encountered in racially segregated and low-resource settings will affect the ability to engage in or benefit from interventions. However, as explained by Brownson et al. (24), the ability to realize the potential of implementation science to improve equity rests in addressing major challenges inherent in the way this field has evolved. They outline these challenges and offer recommendations for addressing them. The challenges are described below.

One set of challenges stems from limitations of the evidence base available to support translation to populations. This evidence base was developed primarily in health-care settings; has limited representation of racial/ethnic minority or low-resource populations; focuses largely on downstream, individually focused interventions without consideration of SDOH; and is not systems oriented. Challenges encountered in attempting to adapt conventional EBIs may lead to the conclusion that new, more relevant interventions must be developed from scratch, involving more time and new resources. This may be particularly important for interventions to be delivered in community settings that are not designed to provide health care but offer broader access to priority populations than health-care settings do (101). Brownson et al. (24) also identify implementation science challenges related to gaps in available methods and measures of sufficient breadth and depth to account for historical, structural, and other contexts. Although implementation science offers new study designs and the potential to measure equity-focused considerations at each stage of the intervention process, more systematic development and vetting of equity-focused measures are needed.

## CHALLENGES AND OPPORTUNITIES GOING FORWARD

This review is written from a US perspective and that of a US-born Black scholar who has focused on obesity and health equity issues for decades. It assumes that health equity in obesity is an achievable goal—one that depends in part on the ability to solve the epidemic overall but requires targeted efforts directed to populations of color and low-resource communities. The challenges for accomplishing this goal have increased because of the coronavirus disease 2019 (COVID-19) pandemic, which has moved the baseline for achieving equity in an adverse direction. For example, numerous and disproportionate deleterious effects of the pandemic on Black Americans have been documented and are linked to structural racism—job loss; inability to social distance when living in relatively crowded conditions, to afford to stay home, or to meet the demands or costs of childcare created by school closings; and the added stress of coping with these effects (20). The higher prevalence of obesity in Black and other marginalized communities has been associated with COVID-19 morbidity and mortality. Hence, COVID-19 has greatly increased the urgency of finding pathways to eliminate obesity disparities.

On the other hand, some progressive US government responses to COVID-19 have now created unparalleled opportunities to address low income as a core SDOH, mainly through new income supports for families with children, increases in SNAP benefits, and changes to other nutrition-assistance programs. If made permanent, these responses could improve standards of living for households with low incomes. These government actions also create opportunities for high-impact research to monitor effects on nutrition, obesity, and overall health and well-being for children and families. Studying implementation of these new policy experiments or advances will be critical to assessing equity effects, for example, to determine whether universal school meal eligibility will differentially affect schools with fewer resources or those serving predominantly racial/ethnic minority children. Assessing implementation of other aspects of COVID-19 recovery at the community level will also be of interest from an equity perspective.

The recommendations for intentionality and adoption of a health equity lens pose professional, personal, and political challenges. As a nutrition researcher or practitioner, the question will arise as to how a focus on equity, with its inherent complexity and inevitable political overtones, affects academic career advancement and self-actualization. On a personal level, the implied moral responsibility to consider equity issues may be challenging, depending on one’s world view or value system and racial/ethnic or social class identity. Political challenges may arise on the basis of regional culture related to the sensitivity around open discussion about race, poverty, and societal versus personal responsibility.

Addressing racism is a core political, and moral, dilemma in US history, and whether it can be mitigated or reimagined in US society is an ongoing question. In this respect, the climate for openly addressing racial justice issues has changed due to a tipping point in political awareness in 2020. Although the issue of police killings of unarmed Black people was not new, the viral dissemination of a video documenting the murder of George Floyd, an unarmed Black man, during an encounter with police in Minneapolis, Minnesota, sparked a new level of public outrage (54). This particular incident and the public drama that ensued followed the widely publicized scholarship and activism related to the 400-year anniversary of Black enslavement in 2019 (62) and was then augmented by professional recognition of the disproportionate harm to Black and Brown communities associated with the COVID-19 pandemic (20, 130). Currently, there are signs that racial equity issues have gained more specificity and traction, including in relation to nutrition and obesity. For example, the importance of an intensified focus on addressing nutrition disparities is emphasized in the future vision for nutrition research (48). However, this improved climate for public and professional discourse may not be sustainable, especially given that the more open discussions about racial justice issues are controversial and highly politicized.

The NIH UNITE initiative to address structural racism in biomedical research and a 2021 NIMHD-sponsored journal supplement on structural racism are examples of this new wave of commitment to addressing racism and discrimination in the United States (111, 126). Many other initiatives related to racial equity are emerging from public and private sector entities including business and philanthropy as well as the nonprofit sector. The response from the research and practice communities to these opportunities will determine their effectiveness and sustainability and will determine future successes. Thus, these developments in no way obviate the need for continued efforts to make the case for racial and socioeconomic equity in society and in health. We need to equip the nutrition and health workforce with skills, tools, and vision. We must expand our skills (e.g., in policy research, measurement of equity inputs to and outcomes of policies and programs, and systems thinking) and our comfort zones (e.g., with explicit discussions of racism) to embrace and implement the strategies recommended here as well as others that might be identified.

Globally, the epidemic of obesity is also not yet under control, and US efforts should be informed by and aligned with global efforts. The 2019 report of *The Lancet* Commission on Obesity outlines a model for bold action. The commission framed the problem of obesity worldwide as one of three pandemics (undernutrition and climate change are the other two) that constitute a syndemic—concurrent, synergistic pandemics with common drivers and the potential to respond to common solutions (136). The report makes it clear that ultimate solutions to obesity must be global and have direct applicability to equity: Marginalized populations are affected disproportionately by the adverse effects of all three pandemics, and the COVID-19 pandemic must now be added. The policy brief that accompanies the detailed commission report provides examples of double- or triple-win solutions that address at least two of the pandemics (137).

The syndemic approach places obesity in a much broader societal context, opening the door to a wider range of solutions, including changes in public and private sector practices related to food systems, transportation, built environments, and land use. Social and political movements are needed to foster and enable these solutions. Such a triangulation of obesity efforts with those that focus on food insecurity and climate change can be effective in the United States if key actors in these policy arenas join forces.

## CONCLUSION

The adverse impacts of the obesity epidemic on the US population are of continuing concern and are even greater among US racial/ethnic minority and low-resource communities due to societal inequities—including disproportionate impacts of the still-evolving COVID-19 pandemic. These inequities urgently require more focused and more systematic attention in the obesity and health equity research and practice communities. Efforts should be informed by systems thinking, give priority to policy-based solutions with broad population reach, and be targeted to address social determinants of eating and physical activity.

## Figures and Tables

**Table 1:** Examples of environmental context variables that may increase risks of developing obesity and decrease responsiveness to interventions in racial/ethnic minority populations and other priority populations

Environment	Food-related variables	Physical activity–related variables
Physical environments that influence inequities in access to and uptake of healthy eating and physical activity options	Limited access to full-service supermarkets	Heavy traffic
	Numerous fast-food outlets	Poor air quality
	Prominent advertisements for high-sugar, high-fat foods inside of stores and outdoors	Lack of pedestrian and cycling pathways
	Limited availability of fresh fruits and vegetables	Concern about crime
	Provision of high-fat, high-sugar foods in schools, workplaces, and other community settings	Limited access to high-quality parks and recreation centers
	Lack of public transportation	Lack of safe and appealing school playgrounds
Economic environments that influence inequities in access to and uptake of healthy eating and physical activity options	Unemployment or unstable employment	Costs of private gyms
	Low income and few opportunities for wealth building	Marketing of digital devices and other sedentary forms of entertainment
	Costs of healthier foods and promotion of less healthy foods at low cost	Limited local investment in parks and recreational facilities
	Limited funds available for school meals	Lack of funds to hire trained physical education teachers in schools
	Prominence of fast-food and soft drink companies as employers or funders of scholarships and community events and projects	Soft drink and fast-food promotions by prominent Black athletes
	Cost of supervised preschool and after-school childcare	Cost of supervised preschool and after-school childcare
Policy/political environments that influence inequities in food and physical activity options	Housing policies	Transportation policies
	Federal and related nutrition and income assistance and food policies	Urban and rural development policies
	Regulations on food advertising	Environmental policies
	Workforce and labor policies	Workforce and labor policies
	School wellness policies	School wellness policies
Sociocultural environments that influence access to and uptake of healthy eating and physical activity options	High-fat, high-sugar foods in traditional cuisine	Cultural norms related to physical activity and the importance of rest
	Childcare and food-related responsibilities of women	Lack of social support or role models for active living
	Caregiver beliefs and feeding practices	Fears about personal safety or child safety
	Body image and perceptions that relatively large body size is culturally acceptable or normative	Gender norms about appropriate physical activity
	Screen time and exposure to food advertising in multiple media channels	Screen time and reliance on TV and digital devices for entertainment

Table adapted from Reference 91. Data from References 15, 39, 55, 56, 70, 74, 84, 91, 98, 123, and 135.

**Table 2:** Selected frameworks to facilitate systematic approaches to equity-focused obesity efforts

Framework	Description
Getting to Equity in Obesity Prevention Framework	Facilitates the process of identifying potentially synergistic combinations of policy, systems, and environmental change interventions to increase options for healthy eating and physical activity or to decrease factors that work against these options as well as identifying strategies to address social needs and community capacity. Associated tools (supplemental files) include definitions of terms, a food-related example of the Centers for Disease Control *Practitioner’s Guide for Advancing Health Equity* (28), examples of logic whereby potential intervention approaches might vary according to contexts, and a tool for assessment of equity considerations in research proposals (87).
Council on Black Health (formerly the African American Collaborative Obesity Research Network) Expanded Obesity Research Paradigm	Uses a people-oriented lens to consider historical and social factors, influences of culture and mind-set, and environments to navigate in relation to healthy eating and physical activity and weight control (90). An accompanying community-centered view of influences on eating, activity, and body weight is designed to guide community-engaged discussions of these issues (56). Also see https://councilbh.org/research-frameworks/.
Taxonomy of Disparities Interventions in Health Care Settings	Provides a taxonomic categorization of elements of health equity research conducted in health-care settings in terms of strategies and tactics used to reduce racial and ethnic disparities in care through changes in behavior, systems, or services and through interventions directed to providers, patients, systems, organizations, and communities or at the policy level (36).
Tool Kit of Adaptation Approaches	Offers a typology of cultural adaptation approaches of behavior change interventions to improve minority health by intervention stages and contexts and decision tools for selecting adaptations across intervention stages (41).
Division of Community Health Twin Approach to Health Equity	Gives guidance for and illustration of the twin or dual approach to health equity in which public health programs simultaneously address the needs of the whole population and population subgroups at high chronic disease risk (32). Illustrations in a two-page fact sheet include healthy eating and active living examples and one for clinical and community linkages (31).
Centers for Disease Control Practitioner’s Guide for Advancing Health Equity	Includes guiding questions to help make the case for why a proposed intervention poses health equity issues, key factors to consider for program design and implementation from a health equity perspective, and the identification of potential barriers or unintended consequences as well as opportunities to maximize impact, needed resources, and potential partners. Illustrations in the guide cover five categories of food-related interventions and six categories related to physical activity (28).

## RACE VERSUS RACISM

Race is primarily a sociopolitical construct that characterizes differential access to power and resources in a society. In contrast, racism is “an organized social system in which the dominant racial group, based on an ideology of inferiority, categorizes and ranks people into social groups called ‘races’ and uses its power to devalue, disempower, and differentially allocate valued societal resources and opportunities to groups defined as inferior” (145, p. 106). This organized social system, developed and maintained at the level of policies and practices in the public or private sector, is referred to as structural racism. Interpersonal racism relates to racially biased or discriminatory actions of individuals. Internalized racism refers to self-perceptions of populations who are the targets of racism, that is, self-derogating beliefs among people of color about themselves or others in the same group (73), and can also refer to internalization of racist concepts and values among people in both dominant and marginalized groups. Cultural racism is the “instillation of the ideology of inferiority/superiority (white privilege) in the values, language, imagery, symbols, and unstated assumptions of the larger society” (145, p. 110) and may lead to both conscious and unconscious interpersonal and internalized racism, including within the health-care system.

## TARGETING AND TAILORING

Adapting Kreuter’s (81) distinctions in relation to individually oriented interventions, targeting refers to directing a policy or program to a specific demographic group. This can be in the context of a universal intervention, that is, a policy or program intended to reach an entire population or population segment, or an intervention that focuses specifically on a priority population such as low-income households or a racial/ethnic minority population with high obesity prevalence. Tailoring refers to building in further adjustments or flexibility such that even interventions targeted to a specific population account for intragroup heterogeneity on contextual factors that are relevant to the intervention design or implementation features that could minimize the potential for differential effects.
